# XMRV Discovery and Prostate Cancer-Related Research

**DOI:** 10.1155/2011/432837

**Published:** 2011-06-21

**Authors:** David E. Kang, Michael C. Lee, Jaydip Das Gupta, Eric A. Klein, Robert H. Silverman

**Affiliations:** ^1^Glickman Urological and Kidney Institute, Cleveland Clinic, 9500 Euclid Avenue, Cleveland, OH 44195, USA; ^2^Department of Cancer Biology, Lerner Research Institute, Cleveland Clinic, 9500 Euclid Avenue, Cleveland, OH 44195, USA; ^3^Taussig Cancer Center, Cleveland Clinic, 9500 Euclid Avenue, Cleveland, OH 44195, USA

## Abstract

Xenotropic murine leukemia virus-related virus (XMRV) was first reported in 2006 in a study of human prostate cancer patients with genetic variants of the antiviral enzyme, RNase L. Subsequent investigations in North America, Europe, Asia, and Africa have either observed or failed to detect XMRV in patients (prostate cancer, chronic fatigue syndrome-myalgic encephalomyelitis (CFS-ME), and immunosuppressed with respiratory tract infections) or normal, healthy, control individuals. The principal confounding factors are the near ubiquitous presence of mouse-derived reagents, antibodies and cells, and often XMRV itself, in laboratories. XMRV infects and replicates well in many human cell lines, but especially in certain prostate cancer cell lines. XMRV also traffics to prostate in a nonhuman primate model of infection. Here, we will review the discovery of XMRV and then focus on prostate cancer-related research involving this intriguing virus.

## 1. Introduction

The retrovirus, xenotropic murine leukemia virus-related virus (XMRV), has generated both interest and debate within the scientific community and also among physicians, patients, and those concerned with maintaining the safety of blood and tissue banks around the world (reviewed in [1–3]). Its discovery was based on the hypothesis that viral infections might contribute to hereditary prostate cancer [4]. Currently, seven types of viruses (HPV, EBV, HHV-8, HTLV-1, HBV, HCV, and MCV) are established etiologic agents of different types of human cancers [5, 6]. While there is also evidence for the presence of viral infections in prostate cancer, including BKV [7], HPV [8, 9], HCMV [10], and EBV [11], thus far there is no compelling evidence that links viral infections to this disease. However, family history is a risk factor for prostate cancer, and in 2002, a combined positional cloning and candidate gene approach mapped a hereditary prostate cancer susceptibility locus, *HPC1* at 1q24-25 [12, 13], to the gene encoding the antiviral protein, RNase L [14]. While several studies have described a link between *RNASEL* and hereditary prostate cancer [14–18], other studies have been unable to confirm the association [19–22]. RNase L is one of the principal antiviral proteins in innate immunity [23]. Type I interferons produced during viral infections induce the pathogen recognition receptors, OAS1 to 3, which produce 2′,5′-oligo(rA) from ATP in response to viral double-stranded RNA. RNase L is present in most mammalian cell types and is activated upon binding to 2′,5′-oligo(rA), thus blocking viral infections by means of RNA degradation [24]. Many different types of viruses are susceptible, in particular viruses with single-stranded RNA genomes, including the retrovirus HIV-1 [25]. The mapping of *HPC1* to *RNASEL* and the invention of a global viral DNA microarray (aka virochip) provided the impetus and means for renewing the search for viruses in prostate cancer [26]. 

**Figure 1:**
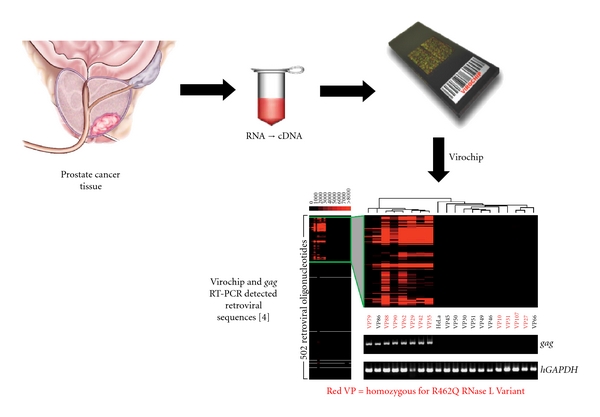
XMRV discovery in prostate cancer study [4]. Human prostate cancer tissues were collected in the operating room at the Cleveland Clinic and used to isolate RNA at either the Cleveland Clinic or at UCSF. At UCSF, RNA was used to synthesize labeled cDNA, virochips were probed, RT-PCR was performed for *gag* sequences, and XMRV cDNAs were sequenced. The RNase L genotypes were determined at the Cleveland Clinic (also the site of the IHC and FISH experiments, not shown). A hybridization pattern typical of a *γ*retrovirus was obtained almost exclusively from patients with the RNase L QQ genotype (red bands and VP codes for QQ patients). RNase L RQ and RR genotypes are shown in black VP codes.

**Figure 2:**
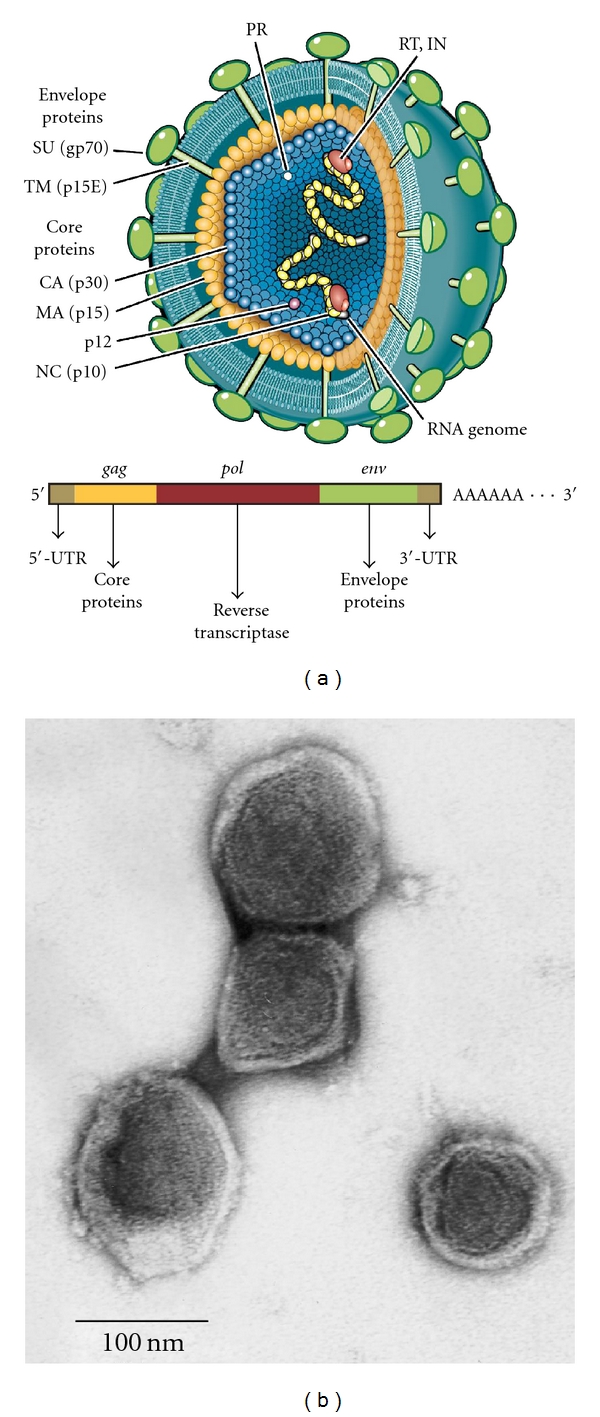
XMRV structure and morphology. (a) Structure of xenotropic murine leukemia virus-related virus showing viral core proteins from the *gag* gene (matrix (MA), capsid (CA), (IN), and nucleocapsid (NC) and p12); from *pol* (protease (PR), reverse transcriptase (RT), and integrase (IN)) and the envelope proteins (surface subunit (SU) and transmembrane subunit (TM) from *env*). Viral particles contain a lipid bilayer envelope and two RNA genomes. (b) Transmission electron microscope image of XMRV (courtesy of Dr. John Hackett, Jr., Abbott Diagnostics, Abbott Park, IL).

**Figure 3:**
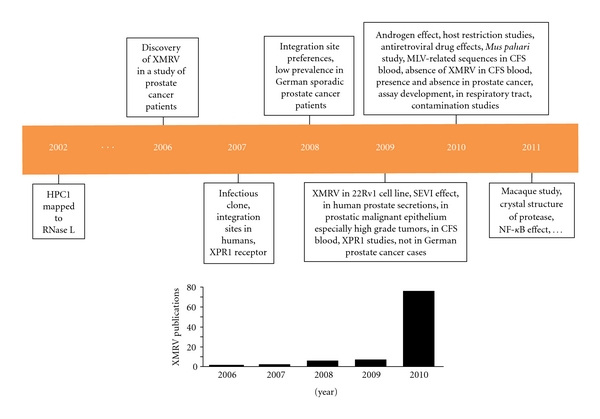
Timeline of XMRV research. Highlights of XMRV studies are shown, including many of the investigations discussed in this review. The mapping of *HPC1* to *RNASEL* was reported in 2002 [14] which led to the discovery of XMRV in 2006 using virochip technology [4]. In 2007, the first infectious clone of was constructed by fusing two overlapping cDNA from prostate cancer patient VP62 [30]. In addition, XPR1 was identified as the receptor for XMRV and the first integration sites in humans were reported [30]. In 2008, additional integration sites were mapped using human prostate cancer tissues [35]. A very low prevalence of XMRV was reported in sporadic prostate cancer patients in Germany [36]. In 2009, XMRV was identified in the human prostate cancer cell line 22Rv1, which had been repeatedly implanted and grown in mice [33]. In addition, a report of XMRV in prostatic malignant epithelium that correlated with tumor aggressiveness appeared [31]. The same year, a study using multiple methods of detection, including PCR, a serology assay for Env and isolation of live virus, showed XMRV in blood of CFS-ME patients, with much lower rates in healthy controls [37]. Studies into XPR1 function and specificity were reported between 2008 and 2010 [34, 38–41], including a study showing that whereas most laboratory strains of mice were resistant to infections, wild mice were susceptible [39]. A study from Germany that used PCR and antibody detection found no evidence of XMRV in prostate cancer [42]. In 2010, the androgen stimulatory effect on XMRV transcription and replication was reported [32, 43]. Host restriction factors, such as APOBEC3G and tetherin, were found to be active against XMRV [44–48]. Antiretroviral drugs were screened and some found to potently inhibit XMRV replication in cell culture [48–50]. The Asian mouse, *Mus pahari*, was exploited for studies on *in vivo* infection [50]. MLV-related sequences were found to associate with CFS-ME [51]. Meanwhile, several other studies, based on PCR and serology, failed to detect XMRV in CFS-ME (e.g., [52]). Two studies confirmed XMRV infections of prostate cancer patients [53, 54] while other studies failed to detect XMRV in prostate cancer patients in the US [55, 56]. Several assays for the detection of XMRV, including a high-throughput automated assay for antibodies against XMRV proteins, were reported [57]. XMRV was reported at a prevalence of almost 10% in immunosuppressed patients with respiratory tract infections in Germany [58]. Papers were published on laboratory contamination with mouse DNA that confounded the search for XMRV in humans [56, 59–62]. In early 2011, a study on XMRV in a non-human primate model showed wide-spread, persistent infection, including the prostate [63]. The crystal structure of the XMRV protease was published [64]. Finally, stimulation of XMRV transcription by proinflammatory cytokines through an NF-*κ*B element in the LTR appeared [65].

The realization that an HPC gene encoded an antiviral protein further suggested the possible involvement of viral infections in prostate cancer. To test this hypothesis, men with localized prostate cancer were genotyped for the R462Q (1385nt G→A) variant of *RNASEL*. A prior study showed that when homozygous this variant doubled the risk of prostate cancer and was implicated in up to 13% of cases [27]. The RNase L Q variant also has about 3-fold reduced enzymatic activity compared with the wildtype R variant [27, 28]. Following radical prostatectomy, RNA isolated from prostate tumors was converted to labeled cDNA and used to screen for evidence of viral sequences by hybridization to virochips composed of the most conserved sequences of all known human, animal, plant, and bacterial viruses [4, 26, 29]. Because the array contained highly conserved sequences within viral nucleic acids, it can detect viruses not explicitly represented. These studies identified the presence of a *γ*retrovirus in 8 (40%) of 20 RNase L R462Q homozygous prostate cancer tissues, and in just 1 (1.5%) of 66 tissues that harbored at least one copy of the wildtype allele (Figure 1). Three XMRV genomes were completely sequenced and were found to share >98% nucleotide and >99% protein sequence identity. Partial sequences were obtained for another six XMRV strains. XMRV is more closely related to the xenotropic and polytropic than to the ecotropic murine retroviruses. XMRV is a canonical *γ*retrovirus, with *gag*, *pro-pol,* and *env* genes, and is not closely related to any endogenous human retroviral (HERV) elements (Figure 2). In addition, XMRV sequences are not present in any human genomic sequences that have been reported to date. A complete provirus clone for XMRV strain VP62 produced infectious virus in LNCaP or DU145 cells [30, 31]. XMRV is able to vigorously infect these and some other cell lines, in particular some prostate cancer cell lines [30, 32–34], allowing for basic virology studies in cell culture system to be conducted. A timeline of XMRV research shows that there was a lag prior to 2010 when a large increase in research papers on the subject appeared in peer-reviewed scientific journals (Figure 3).

## 2. Evidence for and against XMRV in Prostate Cancer

The possibility of laboratory contamination was carefully considered in the XMRV discovery paper in which several lines of evidence supported genuine human infections [4]. First, XMRV was detected using (DNase-treated) RNA directly isolated from fresh frozen, primary human prostate tumor tissues that were not placed in cell culture nor were these human samples exposed to any cultured cell products or cell culture reagents. Second, the extent of sequence variation between the different *gag* and *pol* sequences from different prostate cancer patients was greater than Taq polymerase error rates which range from 10^−6^ to 10^−4^ (see [66] and references therein). These finding suggested natural sequence diversity consistent with independent acquisition of XMRV infections by humans. Third, fluorescence in situ hybridization (FISH) identified XMRV nucleic acid in a small number of stromal cells in tumor-bearing prostate tissue. Fourth, a similar small number of Gag-positive stromal cells were detected in prostate tumor tissues using monoclonal antibody against spleen focus-forming virus Gag with an enhanced alkaline phosphatase red detection method. Fifth, no mouse GAPDH DNA sequences were detected in any of the radical prostatectomy samples providing evidence against contamination from any mouse-derived sources. Finally, XMRV was predominantly restricted to RNase L QQ prostate cancer cases. Therefore, both PCR based and non-PCR evidence supported genuine infection of humans.

Recently, however, the human origin of XMRV has been questioned based in part on the near sequence identity of XMRV strain VP62, isolated using human prostate cancer tissue, with XMRV present in a human prostate cancer cell line, 22Rv1 [33]. The 22Rv1 cells were derived in the 1990s at Case Western Reserve University from a human prostate cancer xenograft by serial passage in mice after castration-induced regression and relapse, raising the possibility that the virus in those cells originated from the mice rather than the patient [67]. A recent study shows that variation between XMRV sequences in the 22Rv1 cell lines exceeded that of XMRV sequences isolated from human specimens, leading the authors to propose that XMRV might not be an authentic human pathogen [59]. In addition, a recent study concludes that the XMRV in 22Rv1 cells originated from two MLV genomes present in the mice used to passage the xenografts ([45], 18th Conference on Retroviruses and Opportunistic Infections).

Additional evidence in favor of genuine infections, including some of the same prostate cancer patients as in the discovery study [4], was provided by XMRV integration site mapping experiments [30, 35]. DNA isolated from human prostate tissues in a PCR-amplicon-free clean room were sent to UCLA and used to precisely map and sequence XMRV integrations sites in nine separate prostate cancer patients [30, 35]. In these prostate cancer tissues, there was a greater tendency for XMRV to integrate near cancer-related genes, microRNA genes, common fragile sites, and cancer breakpoints in comparison to XMRV integration sites in DU145 prostate cancer cells infected in the lab [35]. Those results suggested an *in vivo* selection process for XMRV integrations in certain genes. The viral integration sites in human prostate DNA started precisely after the end of the right-side LTR repeat (5′-*⋯*CTTTCA-3′) demonstrating correct integration had occurred and ruling out artifactual fusion. These experiments also effectively ruled out direct mouse DNA contamination as a source of the XMRV sequences because LTR sequences were fused to human, not mouse, DNA. However, two of the integration sites are identical to XMRV integration sites obtained with XMRV-infected DU145 cells used in the same studies [35, 68, 69]. Therefore, it remains to be confirmed whether these two sites, and the other 12 XMRV integration sites, originated from the patients or from cells infected in the laboratory.

Three independent studies supported XMRV infections of prostate cancer patients at prevalence rates in the range of about 10 to 28%. A study from the University of Utah and Columbia University produced evidence for XMRV infection of prostatic malignant epithelial cells *in vivo* [31]. Immunohistochemistry (IHC) performed on 233 prostate cancer specimens and 101 controls with benign prostatic hyperplasia showed protein staining for XMRV in 23% of cancer cases and 4% of controls. IHC was performed using a polyclonal antibody raised in rabbit against whole virus XMRV (produced in 293T cells—a human kidney epithelial cell line). The presence of antibodies against host-derived proteins among the anti-XMRV antibodies is a limitation of this approach and raises questions about whether the signals detected originated from XMRV infections. Quantitative PCR data from the same study detected XMRV DNA in 6.2% of prostate cancer and 2.0% of control specimens, much lower percentages than through IHC. XMRV associated with higher Gleason Index of prostate cancer but there was no correlation with the R462Q RNase L variant [31]. This report suggested that XMRV is a possible etiologic agent for prostate cancer, and not just a passenger virus.

An investigation at Emory University confirmed the presence of XMRV in men with prostate cancer by utilizing three methods, a novel serum-based assay for neutralizing antibodies against XMRV, nested PCR for *env* sequences, and FISH [53]. The serologic assay detected neutralizing antibodies in 11 of 40 prostate cancer cases (27.5%). Among 20 *RNASEL* QQ patients, 8 (40%) had neutralizing antibody against XMRV, in agreement with the original report of an association of XMRV infection with the QQ genotype [4]. FISH showed XMRV infection in 5–8% of stromal cells of positive cases, and none in epithelial cells [53]. All three methods were in agreement for 5 XMRV positive cases and 2 XMRV negative cases.

Most recently, a study at Baylor University detected XMRV in 32 of 144 (22%) men from the southern US with prostate cancer using a nested PCR assay for the *env* gene [54]. Patients were more likely to score positive for the presence of XMRV in both tumor and normal tissue than in either type of tissue alone. However, there was no correlation between the presence of XMRV and either the RNase L genotype or clinical parameters of disease. The presence of XMRV in normal tissues suggested that infection might precede prostate cancer. 

In support of the presence of XMRV in some prostate cancer patients, a follow-up study at the Cleveland Clinic detected XMRV RNA by nested and quantitative RT-PCR of *env* RNA in expressed prostate secretions of 4 of 32 unselected prostate cancer cases [70]. These findings suggested that XMRV might be present in human semen. However, another study using nested RT-PCR of XMRV *gag* RNA failed to detect XMRV in seminal plasma from HIV-1-infected men in the Netherlands, although some of these subjects were using antiretroviral drugs [71]. 

Several research efforts have either failed to detect XMRV at all, or demonstrated very low prevalence of infection. In the first of several European studies, an investigation in Hamburg, Germany found XMRV RNA by nested RT-PCR in only one of 87 cases in nonfamilial prostate cancer and one of 70 samples from a control population of men with benign prostatic hyperplasia [36]. Both patients contained at least one copy of the wildtype R462 RNase L allele. A study from Berlin failed to detect XMRV in 589 prostate cancer tissues and 146 prostate cancer serum samples utilizing nested PCR for* gag *DNA, RT-PCR for *gag *RNA, and serology assays for antibodies against XMRV Gag and Env. *RNASEL* status was examined in 76 patients in which 12.9% were QQ [42]. Further studies from Europe demonstrated similar negative results. Using PCR, a study of prostate cancer patients in Ireland found no evidence of XMRV DNA by PCR in 139 peripheral blood mononuclear cell (PBMC) samples nor in prostate tissues of seven *RNASEL* QQ prostate cancer cases and two RQ cases, although the QQ genotype was associated with more aggressive disease [72]. A Dutch study of tissue specimens for 74 sporadic prostate cancer patients showed low detection (1 in 600 to 7,000 cells) in only three cases (4%) using RT-PCR for XMRV *integrase* sequences [73]. An international collaborative study centered in the UK utilized nested PCR with *gag* leader primers on DNA extracted from formalin-fixed and paraffin-embedded (FFPE) prostate cancer tissues from the UK, Thailand, and Korea [60]. XMRV-like sequences were detected in 14/292 UK prostate cancers, 5/139 Korean samples, and 2/6 specimens from Thailand. However, upon sequencing, some amplified DNA fragments contained the 24 nt deletion upstream of the *gag* ATG start codon while other amplicons more closely resembled polytropic MLV. Because these results suggested contamination with mouse DNA, a single PCR assay for intracisternal A-type particle (IAP) LTR sequences and a TaqMan qPCR assay for mouse mitochondrial cytochrome oxidase, *cox2*, sequences were performed. These assays are highly sensitive due to large numbers of copies per mouse cell. The presence of the PCR products using XMRV *gag* leader primers in the human DNA samples was completely concordant with IAP sequences, the more sensitive of the two assays for mouse DNA contamination. Therefore, all “XMRV positive” samples were also contaminated with mouse DNA contamination. 

Within North America, negative results have also been reported. In Mexico, XMRV RNA was assayed by nested RT-PCR with only a single positive out of 75 controls with an RNase L RR genotype, and no positives among 55 prostate cancer patients, none of which were QQ genotype [9]. An investigation of different bacteria and viruses in prostate cancer found no XMRV DNA in 200 patients using nested PCR to *gag* [74]. More recently a report that included some of the same authors published an absence of XMRV in over 800 specimens using both RT-PCR and IHC [55]. A duplex PCR assay was used on DNA from 161 prostatic adenocarcinomas in which XMRV *gag* sequences were coamplified with a host gene, but no XMRV was detected. The assay was capable of detecting DNA from a single XMRV infected cell, 22Rv1, in the presence of a large excess of human DNA. In addition, 596 prostate cancers and 452 benign prostate tissue specimens were screened by IHC and all were negative. Recently, prostate tissue DNA from US patients with intermediate- to advanced-stage prostate cancer were tested by PCR assays for XMRV and MLV variants [75]. In three of 162 cases (1.9%), XMRV DNA was detected and sequenced. These samples were negative for mouse mitochondrial DNA using a highly sensitive assay, ruling out contamination from mouse DNA. There was no association with the RNase L QQ variant, plasma was negative for viral RNA by RT-PCR and all 162 patients were negative by a Western blot assay for antibody. The authors concluded that there was no association of XMRV or MLV variants with prostate cancer. However, they also concluded that there was a distinctive XMRV strain in 3 cases, demonstrating a broader diversity in this family of viruses while supporting the case for human infections. A recent study of 110 prostate cancer cases and 40 benign or normal prostate tissues from the Midwestern US concluded no association with XMRV [56]. Although there were 6 PCR-positive cases for MLV sequences (5 prostate cancer and 1 nonprostate cancer) all of these were positive for mouse mitochondrial DNA suggesting contamination. There were sporadic IHC-positive prostate tissues using anti-XMRV antibody [31] but not with an anti-MLV antibody, and none of the serum samples produced strong neutralization of XMRV infections.

While the reasons for these disparate results have yet to be fully resolved, there are several possibilities. For instance, XMRV may be present at extremely low levels *in vivo* and therefore the virus could be missed. Because a single provirus can be transcribed into large numbers of RNA transcripts, detection of RNA may be more sensitive than that of DNA. Differences in geographical distribution of the virus, patient selection criteria, and methodology (e.g., PCR for *env* versus *gag* and tissue processing—e.g., fresh-frozen tissue versus formalin-fixed and paraffin-embedded tissues) are other variables. In some instances positive findings could be tainted by laboratory or reagent contamination, as is the case for mouse nucleic acids in some preparations of Platinum Taq polymerase (Invitrogen) used in PCR [1, 61]. Three examples of laboratory contamination have recently been published, two in prostate cancer specimens already mentioned [56, 60], the other in CFS-ME [62]. Because of the close relationship of XMRV sequence to sequences in mouse genomes, both extraordinary measures to avoid cross-contamination and ultrasensitive methods for detection of mouse DNA and RNA are necessary.

## 3. The Xenotropic and Polytropic Retrovirus Receptor 1 (XPR1) in Human Prostatic Cells

XPR1 is the cell surface receptor and determinant of viral infectivity for XMRV, X-MLVs, and P-MLVs [30, 34, 38, 39, 76–78]. It is a 696 aa protein with eight putative transmembrane domains and four putative extracellular loops (ECL1–4) [76–78]. Despite a common receptor, XMRV has host range and receptor requirements that differ from mouse X/P-MLVs, suggesting adaptations in humans or in intermediate hosts. Residues K500 and T582 in XPR1 ECL3 and ECL4, provide equivalent receptors for X/P-MLV, but not in the case of XMRV [40]. In addition, mouse X-MLV is able to infect all mammals, but XMRV is unique in being restricted in gerbil and hamster cells [40]. There are at least six functionally distinct variants of the XPR1 receptor with varying abilities to support entry by X-MLVs and P-MLVs [41]. While it is unknown whether XMRV found in humans was transmitted directly from infected mice, direct transmission could be reflected in the geographical distribution of virus and/or receptor type in mice, as well as in the worldwide distribution of prostate cancer cases. Interestingly, the most permissive *Xpr1* receptor allele, *Xpr*1^*sxv*^, is found in areas of high prostate cancer incidence such as the United States, while the most restrictive allele, *Xpr*1^*m*^, is found in low tumor rate areas such as Japan and Eastern Europe [38]. 

XPR1 RNA was shown to be present in human prostate stromal fibroblasts but absent in prostatic epithelial and smooth muscle cell lines [79], consistent with some previous findings that XMRV viral antigens are present in prostatic stromal fibroblasts of prostate cancer patients [4, 53]. However, prostate cancer cells of epithelial origin express XPR1 and are susceptible to XMRV infection [34]. XMRV was able to infect, at low levels, cells that did not express detectable levels of XPR1 RNA, suggesting an alternative pathway of infection [79].

## 4. Enhancement of XMRV Infectivity by Fibrils of Prostatic Acid Phosphatase Fragments

Prostatic acid phosphatase is the predominant protein in human semen, and fragments of this protein form positively charged amyloid fibrils that significantly increase HIV-1 infectivity [80]. These fibrils, aka “semen enhancers of virus infection” (SEVI), capture virus particles and greatly increase viral attachment and entry via cell surface receptors by neutralizing negative-charge repulsion between the HIV-1 virion and the cell surface [81]. SEVI has also been shown to enhance XMRV infections via the XPR1 receptor in human prostate cancer cell line DU145 [70]. SEVI enhanced XMRV attachment and fusion while lowering the threshold for infectivity by up to 4,000-fold. XMRV infectivity was enhanced by SEVI in a wide range of different cell types, including primary prostatic epithelial and stromal cells [70]. XMRV infectivity in cell culture was similarly enhanced by human semen, and this was most pronounced at low viral doses. These results, and the presence of XMRV RNA in prostate secretions, suggest sexual transmission as a potential biological mechanism for viral spread, although confirmation by seroprevalence and other epidemiologic studies is required before such a conclusion can be made. However, XMRV infection of rhesus macaques by the IV route showed that the virus traffics to and infects prostate epithelium within 6 to 7 days of infection [63]. In addition, a separate study, reported in this issue by Sharma et al., demonstrates that XMRV infects the reproductive tracts of both male and female macaques further suggesting the possibility of sexual transmission [82].

## 5. Host Restriction of XMRV in Prostate Cancer Cell Lines

Many host restriction factors are IFN regulated and collectively contribute to the IFN-induced antiviral state [83]. For example, IFNs induce OAS proteins that produce the 2′–5′-oligo(rA) activators of RNase L. As a result, RNase L suppresses replication of a wide range of viruses in cells exposed to IFN [24]. Sustained activation of RNase L also drives cells into apoptosis, a potential antitumor cell as well as an antiviral mechanism [84, 85]. Therefore, *RNASEL *mutations could contribute to prostate cancer by allowing clonal expansion of mutant cells that have escaped apoptosis and/or by allowing persistent infection by oncogenic viruses. Accordingly, reduction in RNase L levels by RNAi decreased the IFN antiviral effect against XMRV in DU145 cells [30]. However, in another study decreasing levels of RNase L using an RNAi approach did not enhance XMRV replication in 293T cells [32]. 

The human APOBEC3 family of cytidine deaminases includes seven members (A3A to H) encoded on chromosome 22 as a gene cluster [86]. A3G causes cytidine deamination in viral minus strand DNA causing G→A hypermutation in the coding strand thus potently inhibiting infectivity and spread of HIV-1 lacking Vif protein [87, 88]. XMRV lacks an inhibitor such as Vif and is highly susceptible to inhibition by A3G [44–47]. Accordingly, there was characteristic G-to-A hypermutation of XMRV DNA in T cell lines H9 and CEM that express A3G and A3F, but low levels of such mutations in prostate cancer cell lines, LNCaP, DU145, and 22Rv1, that lack A3G [45]. Primary prostatic stromal fibroblasts varied in expression of A3G mRNA from undetectable to moderate levels [46]. These findings suggest that prostate cancer cells and stromal fibroblast might provide a favorable environment for XMRV infection and replication *in vivo*. 

Groom et al. investigated the effects of the murine protein Fv1 and the TRIM5*α* family of proteins [44]. XMRV was restricted by Fv1^n^ and Fv1^b^ but not restricted by any of 13 TRIM5 proteins tested. However, XMRV was highly susceptible to inhibition by the IFN-inducible protein, tetherin, that links viruses to the plasma membrane during budding [89].

## 6. Androgen Regulation of XMRV

Transcriptional control of the XMRV genome is mediated by *cis*-acting elements in the 5′-LTR U3 region. This 390-nucleotide segment contains the promoter and enhancers, as well as two glucocorticoid response elements (GRE). Other examples of GREs respond to glucocorticoids, mineralocorticoids, progesterone, and androgens. Furthermore, tropism studies of cultured cells suggest a role for the androgen receptor in promoting XMRV replication [30, 32, 34, 43]. XMRV was readily able to spread and replicate in androgen receptor positive LNCaP cells, but not in various other cell lines that lacked androgen receptor [32]. Dihydrotestosterone treatment of LNCaP cells caused a twofold and threefold increase of XMRV transcription and replication, respectively [43]. Conversely, the androgen inhibitors, casodex and flutamide, inhibited XMRV replication by up to threefold, which suggests that androgen ablation therapy used in prostate cancer treatment could inhibit viral growth [43]. A point mutation in one of the XMRV GREs led to impaired androgen regulation of XMRV transcription and replication [43]. Enhancer elements in the XMRV LTR could impart androgen regulation to integrated host genes, thus potentially contributing to oncogenesis.

## 7. Conclusions

There are a number of potential mechanisms by which a retrovirus could cause prostate cancer. Retroviruses generally transform cells by insertional activation of an oncogene, transduction of a host-derived oncogene, or oncogenesis by a viral protein (e.g., the JSRV Env protein) [90, 91]. *γ*retroviruses, which lack a host-derived oncogene, typically cause cancer by insertion of the LTR near a cellular proto-oncogene leading to its activation. One can, however, envision possible alternative oncogenic mechanisms. For instance, viral infection in stromal cells might alter the microenvironment thus indirectly promoting neoplastic transformation. Infected stromal cells might induce cytokines, chemokines, or growth factors, creating a microenvironment conducive to tumorigenesis [92, 93]. Uncoordinated integration of viral DNA ends is another potential mechanism through which retroviruses may induce genomic alterations. However, it was recently shown that XMRV integration proceeds with high fidelity and involves a coordinated joining of the two viral DNA termini in the host genome flanked by a 4 bp direct repeat of host DNA [68].

XMRV does not have direct transforming activity in standard focus formation assays in fibroblast and epithelial cell lines [94, 95]. However, XMRV did rarely induce transformation of a rat fibroblast line, suggesting an indirect mode of action. Most likely, in order for XMRV to contribute to tumorigenesis through this mechanism, active viral replication with multiple integration events would be required until integration occurred in a cellular oncogene [94]. 

Claims of novel human retroviruses have often been met with considerable skepticism and resistance, earning the moniker human “rumor viruses” and XMRV is certainly no exception. While both PCR and non-PCR based evidence from several different laboratories collectively provide support for infections of some prostate cancer patients with XMRV, or similar viruses, an extreme level of caution is required to avoid laboratory contamination. Finally, only hypothesis-driven research that directly tests for infection and modes of pathogenesis for this virus can answer questions about its importance in disease.
